# The Treatment of Cold Abscess

**Published:** 1893-04-15

**Authors:** 


### ROYAL INFIRMARY, EDINBURGH.

### The Treatment of Cold Abscess.

It is now very generally believed that the chronic or cold abscess is in almost every case a manifestation of the scrofulous condition, and results from the breaking down of a local tubercular focus. It must, therefore, be treated on the same principle as other tubercular affections, by the removal of all diseased parts as thoroughly and completely as possible.

When these slow-forming collections of pus appear in the subcutaneous tissues, as they not unfrequently do, they are of small size, and may be multiple. When, on the other hand, they take origin from bone—for example, the bodies of the lumbar vertebræ, constituting the well-known psoas abscess—they assume large dimensions and irregular shapes.

Clinically they are characterised by an insidious onset, a slow progress, and the absence of the more prominent signs and symptoms of suppuration. Thus, seldom can a definite cause be assigned; the swelling has often attained considerable size before attracting the attention of the patient, and when examined does not present any redness or heat, and causes little or no pain. A soft fluctuating swelling is all that can be made out. The contained pus is thin and watery, with shreds and flakes of lymphy material, and sometimes spicules of bone. The ordinary pyogenic micro-organisms are absent, but the tubercle bacillus may be detected.

From the point of view of treatment, one or two pathological points are worthy of being borne in mind. The interior of the abscess cavity is covered by a distinct, more or less dense lining—the so-called "pyogenic membrane" — composed of closely-packed granulation tissue, the whole of which must be removed before a satisfactory result can be obtained. Again, the gradually increasing pressure and irritation of the accumulating pus tends to thicken the fasciae with which it is in contact, rather than to ulcerate through it, while the pus thus pent up finds its way along the lines of diminished resistance by the sides of the large blood vessels. In this way the abscess cavity may assume a very irregular shape, and may contract important relations not to be forgotten during operative procedures.

The strictest antiseptic precautions must be observed to exclude putrefactive organisms, as the contents of these abscesses afford an excellent nidus for such, and grave consequences follow their introduction.

There are therefore three great objects to be attained in treating a cold abscess, (1) to exclude sepsis, (2) to get rid of the pyogenic membrane, and (3) to avoid injuring important blood vessels. The use of the aspirator, the trochar and canula, or the frequent making of small valvular punctures attain the first and last of these objects, but fail entirely to effect the second. A free antiseptic incision, with frequent dressings, long-continued drainage, and absolute rest admits of the walls coming together and gradually closing the cavity by the union of the granulating surfaces, but the time spent, and the great risk of septic infection at any stage of the treatment are serious objections to this method.

Mr. F. M. Caird, of this hospital, in a considerable number of cases has obtained most satisfactory results by thoroughly removing the pyogenic membrane with the finger nail, and then bringing the raw surfaces together with stitches, with a view to getting healing by first intention.

The patient is anæsthetised, the skin having been purified in the usual way, namely, by being covered for twelve hours with a carbolic towel (1-20), and then thoroughly scrubbed with soda, turpentine, and carbolic. An incision is made just large enough to admit the finger, which is introduced before any of the pus escapes, and with the nail the whole of the membrane is peeled off. When the cavity is too large to be reached from one opening, others are made in the same way, but care is taken that the pus does not escape till all the wall has been cleared. The cavity is then flushed out with a warm saturated solution of boracic acid till the lotion flows out clear. Deep stitches may then be introduced, but not tightened till next day, small capillary drains being left in for twenty-four hours. Before applying the deep dressing, firm pressure is made over the cavity to expel air and bring the walls in contact, and this pressure is kept up by pads of gauze and an elastic bandage. An ordinary antiseptic dressing is applied, and changed the following day, when the drains are removed and the stitches tightened. Splints are employed to secure rest, and subsequent dressings are carried on as may be indicated. This method also fulfils the third condition required, the finger-nail being safer than sharp spoons in the vicinity of important structures.

